# Mutagenic Activity of *Indigofera truxillensis* and *I. suffruticosa* Aerial Parts

**DOI:** 10.1093/ecam/nep123

**Published:** 2011-02-17

**Authors:** Tamara Regina Calvo, Cássia Regina Primila Cardoso, Adriana Candido da Silva Moura, Lourdes Campaner dos Santos, Ilce Mara Syllos Colus, Wagner Vilegas, Eliana Aparecida Varanda

**Affiliations:** ^1^Departamento de Química Orgânica, Instituto de Química de Araraquara, UNESP-São Paulo State University, c.p. 355, CEP 14800-900, Brazil; ^2^Faculdade de Ciências Farmacêuticas, Departamento de Ciências Biológicas, UNESP-São Paulo State University, CEP 14801-902, Araraquara, SP, Brazil; ^3^Department of General Biology, Biological Sciences Center, UEL-Londrina State University, C.P. 6001, CEP 86051-990, Londrina, PR, Brazil

## Abstract

*Indigofera truxillensis* and *I. suffruticosa*, are used as a source of indigo dye and to treat several diseases. The mutagenic activity of the methanolic extracts from aerial parts, glycerolipid, flavonoid and alkaloid fractions of the extract were evaluated by means of *Salmonella/*microsome assays using TA100, TA98, TA102 and TA97a strains. The methanolic extract of *I. truxillensis* showed mutagenic activity in the TA98 strain without S9 while glycerolipid fraction was devoid of activity. The flavonoid and alkaloid fractions of both plants showed mutagenicity. Chemical analysis of flavonoid fractions of *I. truxillensis* and *I. suffruticosa* resulted in the identification of kaempferol, quercetin and their derivatives. The alkaloid fraction of both the species contained indigo and indirubin and indigo was found mainly responsible for the mutagenic activity.

## 1. Introduction

*Indigofera truxillensis* and *I. suffruticosa* (family Fabaceae) are common plants of the Brazilian savannah. The genus *Indigofera* is known to be a rich source of flavonoid glycosides [1, 2] and indigo derivatives (bis indoles) [3] and nitro compounds [4]. *Indigofera truxillensis* Kunth is reported to be antiulcerogenic and antioxidant [5, 6]. *Indigofera suffruticosa* Miller is used as a source of indigo dye and in popular medicine as an antimicrobial, purgative, anti-spasmodic, sedative, diuretic, to treat epilepsy, stomach and urinary diseases, jaundice, ulcers, intermittent fevers, hepatitis, as an antidote for snake venom and bee bites and to stimulate the central nervous system [7]. Recently it was found highly effective in inhibiting growth of solid tumors [8] and showed antibacterial and antifungal activities [9].

Investigation of traditionally used medicinal plants is thus valuable as a source of potential chemotherapeutic drugs and as a measure of safety for the continued use. Plants are used to treat various ailments, however, some medicinal plants can be with serious risks to humans health [10]. The *Salmonella* mutagenicity test (Ames test) identify if any sample provoke the mutation of genetically modified DNA of selected *S. typhimurium* strains and is used worldwide as an initial screening of the mutagenic potential of new chemicals for hazard identification and for the registration or acceptance of new chemicals by regulatory agencies [11, 12].

In spite of the many beneficial actions of plants, it is important to emphasize that some of their constituents can be poisonous to the organism, and metabolism of ingested plants can also generate toxic metabolites. Many carcinogens remain inactive until they are enzymatically transformed to an electrophilic species that is capable of covalently binding to DNA, leading to mutation. For this reason, metabolic activation is considered to be a critical step in mutagenesis [13–16].

Short-term tests that detect genetic damage can provide information needed to evaluate carcinogenic risks of chemicals to humans. The Ames test, recommended for testing the mutagenicity of chemical compounds with potential pharmacological application [12, 17], was used in the present study to evaluate the putative mutagenic effect of the methanolic extracts of *I. truxillensis*, *I*. *suffruticosa*, flavonoid and alkaloid fractions including indigo and indirubin.

## 2. Methods

### 2.1. Chemicals

Dimethylsulfoxide (DMSO), nicotinamide adenine dinucleotide phosphate sodium salt (NADP), d-glucose-6-phospate disodium salt, l-histidine monohydrate, d-biotin, standard mutagens: sodium azide, 2-anthramine, mitomycin C and 4-nitro-*o*-phenylenediamine were purchased from Sigma Chemical Co. (St Louis, MO, USA). All other reagents used for chemical analysis and to prepare buffers and media were from Merck (Whitehouse Station, NJ) and Sigma.

### 2.2. Plant Material

Aerial parts of plants were collected in Rubião Junior, Botucatu city, São Paulo State, Brazil, and authenticated by Prof. Dr Jorge Yoshio Tamashiro. The voucher specimens of *I. suffruticosa* Miller (HUEC 129598) and *I. truxillensis* Kunth (HUEC 131827) were deposited at the Herbarium of the Universidade Estadual de Campinas (Unicamp), Campinas, SP, Brazil.

### 2.3. Extraction and Isolation

Air-dried aerial parts of the plants (1.5 kg) were powdered, extracted with chloroform (CHCl_3_) and methanol (MeOH) successively at room temperature (3 × 72 hours, each solvent). Solvents were filtered and evaporated at 35°C under reduced pressure, affording the CHCl_3_ and MeOH extracts, respectively. *Indigofera truxillensis* yielded 43.0 g (2.9%) of CHCl_3_ and 110.0 g (7.3%) of MeOH extract, while *I. suffruticosa* furnished 18.5 g (1.2%) of CHCl_3_ and 41.3 g (2.7%) of MeOH extract. The MeOH extracts of both *Indigofera* species were fractionated in analogous ways by gel permeation chromatography. An aliquot of the MeOH extract of the each plant (2.8 g) was subjected to column chromatography on Sephadex LH-20 (*∼*130 fractions of 20 ml), using methanol as eluent, flowing at 0.5 ml/minutes. The collected fractions were combined into fractions A–C, after thin layer chromatography (TLC) analysis on silica-gel TLC plates on glass (20 × 20 cm), run with a solvent mixture composed of butanol : acetic acid : water (4 : 1 : 2, v : v : v), visualized by UV light (254 and 365 nm) and then sprayed with diphenylaminoborate/polyethyleneglycol (NP/PEG) reagent or anisaldehyde/sulfuric acid solution to develop the spots [18].

Fraction A, obtained from the MeOH extracts of each species of *Indigofera*, was analyzed by direct injection ESI-IT-MS/MS (electrospray ionization ion trap tandem mass spectrometry), which demonstrated that this fraction contained glycerolipids. Fraction B, denominated the flavonoid fraction, was purified by column chromatography on polyvinylpyrrolidone, eluted with MeOH and MeOH : water (80 : 20, v : v), or MPLC (medium-pressure liquid chromatography) on silica-gel, eluted with EtOAc : MeOH under gradient conditions, followed by semi-preparative reversed-phase HPLC on C-18 silica-gel, eluted with MeOH : water (80 : 20; 60 : 40, v : v). The purification of the fraction B from *I. truxillensis* and *I. suffruticosa* afforded flavonol derivatives of kaempferol and quercetin, respectively.

Fraction C, denominated the alkaloid fractions, obtained from the MeOH extracts of *I. truxillensis* and *I. suffruticosa*, was chromatographed by Sephadex LH-20 column and exhibited bis-indole alkaloids. Where necessary, fractionation of the MeOH extracts was repeated to obtain larger quantities of fractions B (flavonoid fraction) and C (alkaloid fraction). Compounds in fractions B and C were identified by MS (mass spectrometry), 1D and 2D NMR (nuclear magnetic resonance) techniques and confirmed by comparing the physical and spectroscopic/spectrometric data (NMR and MS) with those in the literature.

### 2.4. General Experimental Procedures

NMR spectra were obtained on a Varian Inova-500 NMR spectrometer using the solvent DMSO-*d_6_* with tetramethylsilane as internal standard. Electrospray ionization mass spectrometry was performed in a Fisons VG Platform instrument in the negative mode (45 V). The samples were dissolved in methanol and injected directly into the mass spectrometer through a Rheodyne injector. Acetonitrile was used as solvent and nitrogen was used as the drying gas and for nebulization. The analyses by ESI-IT-MS/MS were performed in a Finnigan (Thermo Finnigan, San Jose, CA, USA) LCQ Deca ion trap instrument equipped with Xcalibur software; samples were dissolved in methanol and infused in the eletrospray ionisation source with a syringe pump. Precoated silica-gel plates with aluminum-backed sheets (Merck) were employed for TLC with detection at 254 and 365 nm followed by color development with NP/PEG reagent or anisaldehyde/sulfuric acid reagent. Sephadex LH-20 columns (25–100 *μ*m, 3.0 (i.d.) × 57.0 and 1.5 (i.d.) × 30 cm, Pharmacia Fine Chemicals), polyvinylpyrrolidone (P–6755, Sigma) and silica-gel SiF254 (0.063–0.200 mm, Merck) were used for column chromatography. The MPLC separations were carried out with a Baeckström apparatus equipped with an FMIQSY lab pump, using a silica-gel column (0.04–0.063 mm, 2.0 (i.d.) × 30 cm, Merck]) Fractions were purified by HPLC in a system equipped with an R401 refractive index detector and with a Phenomenex Luna reversed-phase C-18 column (10 mm, 1.0 (i.d.) × 25 cm, Phenomenex Luna])and Rheodyne injector with a 100 *μ*l sample loop.

### 2.5. S. typhimurium Mutagenicity Assay

It was performed by preincubating test compounds for 20–30 minutes with the *S. typhimurium* strains TA100, TA98, TA97a and TA102, with or without metabolic activation [11]. The S9-mix was freshly prepared before each test with an Aroclor-1254-induced rat liver fraction purchased (lyophilized) from Moltox (Molecular Toxicology Inc.). *Salmonella typhimurium* strains were kindly provided by Dr B. Ames, University of California, Berkeley, CA, USA. Various concentrations of the dry MeOH extract (1.25–7.5 mg/plate), the flavonoids fraction (0.12–2.5 mg/plate), alkaloids fraction (0.12–2.5 mg/plate) and isolated compounds (indigo and indirubin: 0.125–1.0 mg/plate) all dissolved in DMSO, were used. The concentrations used were based on the bacterial toxicity, in a preliminary test. In all subsequent assays, the upper limit of the dose range tested was either the highest non-toxic dose or the lowest toxic dose determined in this preliminary assay. Toxicity was apparent either as a reduction in the number of his*+* revertants or as an alteration in the auxotrophic background lawn. The various concentrations of tested compounds were added to 500 *μ*l of buffer (pH 7.4) and 100 *μ*l of bacterial culture and then incubated at 37°C for 20–30 minutes. Next, 2 ml of top agar was added to the mixture and the whole poured on to a plate containing minimum agar. The plates were incubated at 37°C for 48 hours and the his+ revertant colonies were counted manually. The influence of metabolic activation was tested by adding 500 *μ*l of S9 mixture (4%) in place of the buffer. All experiments were analyzed in triplicate.

The standard mutagens used as positive controls in experiments without S9 mix were 4-nitro-*o*-phenylenediamine (10 *μ*g/plate) for TA98 and TA97a, sodium azide (1.25 *μ*g/plate) for TA100 and mitomycin C (0.5 *μ*g/plate) for TA102. 2-anthramine (0.125 *μ*g/plate) was used in the experiments with metabolic activation with all strains. DMSO served as the negative (solvent) control.

### 2.6. Statistical Analysis

The statistical analysis was performed with the Salanal computer program, adopting the Bernstein et al. [19] model. The mutagenic index (MI) was also calculated for each dose, as the average number of revertants per plate divided by the average number of revertants per plate of the negative (solvent) control. A sample was considered positive when MI ≥2 for at least one of the tested doses and if the response was dose dependent [20–22].

## 3. Results

### 3.1. Phytochemical Analysis

Portions of the MeOH extracts from *I. truxillensis* and *I. suffruticosa* were fractionated by gel permeation on Sephadex LH-20, leading to the collection of fractions A–C. Fraction A from both species contained glycerolipids.

**Figure 1:**
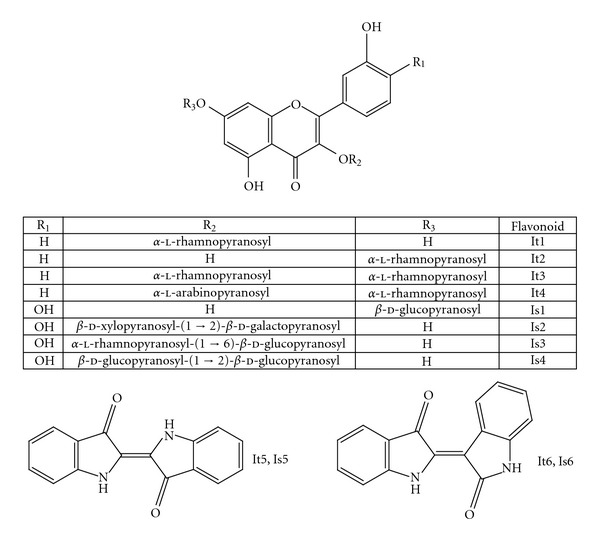
Structures of the compounds isolated from the *Indigofera* species. Fraction B (Flavonoid fraction): *I. truxillensis—*kaempferol 3-*O*-*α*-l-rhamnopyranoside (It1) and kaempferol 7-*O*-*α*-l-rhamnopyranoside (It2), kaempferol 3-*O*-*α*-l-rhamnopyranoside-7-*O*-*α*-l-rhamnopyranoside (It3) and kaempferol 3-*O*-*α*-l-arabinopyranoside-7-*O*-*α*-l-rhamnopyranoside (It4); *I. suffruticosa*—quercetin 7-*O*-*β*-d-glucopyranoside (Is1), quercetin 3-*O*-[*β*-d-xylopyranosyl-(1→2)-*β*-d-galactopyranoside] (Is2), quercetin 3-*O*-[*α*-l-rhamnopyranosyl-(1→6)-*β*-d-glucopyranoside] (Is3) and quercetin 3-*O*-[*β*-d-glucopyranosyl-(1→2)-*β*-d-glucopyranoside] (Is4). Fraction C (Alkaloid fraction): *I. truxillensis* and *I. suffruticosa—*indigo (It5, Is5) and indirubin (It6, Is6).

The purification of fraction B (flavonoid fraction) from *I. truxillensis* yielded the flavonols: kaempferol 3-*O*-*α*-l-rhamnopyranoside (It1, 9 mg) and kaempferol 7-*O*-*α*-l-rhamnopyranoside (It2, 7 mg), kaempferol 3-*O*-*α*-l-rhamnopyranoside-7-*O*-*α*-l-rhamnopyranoside (It3, 21 mg), kaempferol 3-*O*-*α*-l-arabinopyranoside-7-*O*-*α*-l-rhamnopyranoside (It4, 15 mg). The flavonoid fraction from *I. suffruticosa* was also purified, affording the flavonols quercetin 7-*O*-*β*-d-glucopyranoside (Is1, 5 mg), quercetin 3-*O*-[*β*-d-xylopyranosyl-(1→2)-*β*-d-galactopyranoside] (Is2, 10 mg), quercetin 3-*O*-[*α*-l-rhamnopyranosyl-(1→6)-*β*-d-glucopyranoside] (Is3, 20 mg), quercetin 3-*O*-[*β*-d-glucopyranosyl-(1→2)-*β*-d-glucopyranoside] (Is4, 8 mg). Purification of fraction C (alkaloid fraction) from *I. truxillensis* and *I. suffruticosa* gave the same bis-indole derivatives: indigo (It5, 5 mg; Is5, 7 mg) and indirubin (It6, 8 mg; Is6, 5 mg) (Figure 1).

### 3.2. Salmonella Mutagenicity Assay

The MeOH extract as well as the fractions and some isolated compounds were investigated for their mutagenic activity, using the *Salmonella* microsome assay.  Table 1 shows the number of revertants/plate, the SD and the MI values after the treatments with the extracts and fractions of *I. truxillensis*, in the four different strains of *S. typhimurium*, with or without metabolic activation. The MeOH extract was mutagenic to the strain TA98 in absence of metabolic activation (−S9) and in presence of S9 TA98 did not display mutagenicity. This strain detects frameshift mutations in the DNA (target –C–G–C–G–C–G–C–G–). The mutagenic indexes per plate observed for the strain TA98 were higher than the other strains used. Fraction A did not display any mutagenicity and it was not further investigated. Fraction B (flavonoid fraction) showed signs of mutagenic activity to the strain TA98 (−S9 and +S9). The values of the MI varied from 1.1 to 1.9, with a significant dose-dependent effect (*P* ≤ .01). Fraction C (alkaloid fraction) also showed signs of mutagenic activity, with MI 1.7 and *P* ≤ .05. These results suggested that the compounds in the methanol extract that induced mutagenic activity were present in fractions B and C.

Table 2 shows the results obtained with the MeOH extract of *I. suffruticosa* and in spite of the negative results in the mutagenic activity, the MI values are around 2 (TA98-S9), suggesting the presence of compounds potentially mutagenic. For the fractions A, B and C the results are similar to those obtained for *I. truxillensis*. The isolated compounds (from Fraction C) were evaluated and it can be seen in Table 3 that for indigo and indirubin the mutagenicity was positive. A significant increase in the reversion frequency of the TA98 strain was observed for indigo with and without addition of the S9 mixture and for indirubin the mutagenic effect was observed in absence of metabolization. The highest MI value (7.7) was observed for indigo in presence of S9 fraction.

## 4. Discussion and Conclusions

Plants of the genus *Indigofera* are known for their efficacy in popular medicine. Although much work has been done on the pharmacological properties of extracts from species belonging to the genus, no data are available in the literature concerning the genotoxicity of *I. truxillensis* and *I. suffruticosa* that might guarantee the safe use of these medicinal plants.

The present study, mutagenic activity assays with *Salmonella* demonstrated that the methanol extract of *I. truxillensis* induced mutagenic activity in the TA98 strain, flavonoid and alkaloid fractions induced a significant increase in the number of revertants per plate, although MI was <2. The methanol extract and fractions of *I. suffruticosa* also induced a significant increase in the number of revertants per plate and the MI values are around 2, indicating signs of mutagenic activity. The phytochemical investigation showed that the flavonoids in the methanol extracts are kaempferol and quercetin glycosides.

In the case of the flavonoids, despite many results indicating their pharmacological activity and potential benefit to human health [23, 24], several are also described as mutagens [25, 26]. Among flavonoids, flavonols constitute a very important subclass as for as genotoxicity studies are concerned. There are numerous reports on the mutagenicity of compounds belonging to this subclass. Quercetin is known to be directly mutagenic to the *Salmonella* strain TA98 [27–30], whereas flavonoids lacking the adjacent hydroxyl (catechol groups) are innocuous [31]. Kaempferol has only one hydroxyl group in the B ring and is a weak mutagen in both TA98 [27–30] and V79 [32] cells, and this activity is decreased further when the aglycone is bound to glycosidic moieties [33].

The indigoids are natural bis-indoles utilized in dyes [34] and are being studied for medicinal purposes [35]. They are founded in plants [36], mushrooms [37] and human urine [38]. In the human body, indole is a product of the catabolism of tryptophan by gut bacteria and is significantly absorbed. It is oxidized to indoxyl and excreted in the urine as indoxyl (3-hydroxyindole) sulfate. Many alkaloids are known to be genotoxic [39–43]. However, many of these alkaloids have also demonstrated an outstanding pharmacological potential, exhibiting antimicrobial, antiplasmodial and antitumoral activities [44, 45].

In relation to indigo mutagenicity the data are controversial. Herbert et al. [46] assessed the mutagenicity of natural indigo by using the standard procedure for the *Salmonella*/microsome mutagenicity test, as described by Ames. The substance exhibited mutagenicity towards strains TA1538 and TA98 in the presence of S9. On the other hand, Jongen and Alink [47] investigated the mutagenic potential of two natural and seven synthetic, commercial indigo dye products. The natural products showed no mutagenicity in *S. typhimurium* strains TA98 and TA100. The results confirmed the presence of mutagenic activity and the highest values of MI were obtained when indigo was evaluated, suggesting that this alkaloid is the main compound responsible for the mutagenic effect. Natural indigo is a dark blue powder obtained from several plant species, besides the gender *Indigofera* [48]. It is used in Chinese traditional medicine [49] for the treatment of virus infections [50], inflammatory breathing diseases [51] and leukemia [52–54]. The bis indole indirubin is an active ingredient of *Danggui Longhui Wan*, a traditional Chinese medicine used in the treatment of chronic diseases such as leukemia. The antitumoral properties of indirubin appear to correlate with their antimitotic effect. Indirubins were recently described as potent inhibitors of cyclin-dependent kinases [55].

Finally, it may be conclude that mutagenic activity observed in the methanol extract can probably be attributed to interaction between several compounds present in these species and the alkaloid indigo is the main compound responsible for this effect. The positive results in the Ames test for the MeOH extract suggest that the indiscriminate use of homemade preparations of this plant can be dangerous to health. Like synthetic medicines, natural products also need to be evaluated with regard to their pharmacological properties, toxicity, dosage, duration of treatment and safety.

## Figures and Tables

**Table 1:** Mutagenic activity expressed as the mean and SD of the number of revertants/plate in bacterial strains TA98, TA100, TA97a and TA102 exposed to MeOH extract and fractions A, B and C of *I. truxillensis* at various doses, with (+S9) or without (−S9) metabolic activation.

Treatments mg/plate	Revertants/plate in *S. typhimurium* strains							
	TA98		TA97a		TA100		TA102	
	−S9^(a)^	+S9^(b)^	−S9^(a)^	+S9^(b)^	−S9^(c)^	+S9^(b)^	−S9^(d)^	+S9^(b)^
MeOH extract								
0	43 ± 2.7	36 ± 2.0	131 ± 1.5	139 ± 2.5	163 ± 4.2	145 ± 9.5	242 ± 9.2	223 ± 11.9
1.25	55 ± 4.6* (1.3)	41 ± 2.3 (1.1)	143 ± 5.0 (1.1)	131 ± 2.5 (0.9)	184 ± 8.7 (1.1)	151 ± 14.5 (1.0)	271 ± 4.0 (1.1)	219 ± 7.9 (0.9)
2.50	57 ± 1.5** (1.3)	38 ± 7.6 (1.1)	143 ± 3.1 (1.1)	129 ± 10.7 (0.9)	186 ± 7.6 (1.1)	145 ± 3.0 (1.0)	254 ± 1.5 (1.0)	223 ± 11.0 (1.0)
3.75	87 ± 2.0** (2.0)	42 ± 2.0 (1.3)	147 ± 2.1 (1.1)	137 ± 6.8 (0.9)	194 ± 4.0 (1.2)	149 ± 11.9 (1.0)	258 ± 1.0 (1.1)	238 ± 6.7 (1.1)
5.00	77 ± 6.1** (1.8)	45 ± 4.2 (1.3)	148 ± 5.0 (1.1)	142 ± 16.1 (1.0)	180 ± 6.2 (1.1)	141 ± 7.6 (0.9)	264 ± 4.0 (1.1)	228 ± 5.3 (1.0)
7.50	75 ± 5.0** (1.7)	51 ± 3.1(1.4)	159 ± 7.8 (1.2)	134 ± 16.0 (0.9)	173 ± 5.0 (1.1)	140 ± 5.5 (0.9)	280 ± 35.6 (1.2)	249 ± 11.0 (1.1)
Fraction A (glycerolipids)								
0	29 ± 2,1	31 ± 2.7	141 ± 8.0	143 ± 4.0	148 ± 2.5	143 ± 5.0	239 ± 16.7	210 ± 11.1
0.12	38 ± 4.6 (1.3)	31 ± 3.6 (1.0)	143 ± 13.5 (1.0)	137 ± 5.3 (0.9)	163 ± 26.1 (1.1)	155 ± 4.2 (1.1)	267 ± 14.1 (1.1)	235 ± 13.1 (1.1)
0.38	32 ± 5.3 (1.1)	30 ± 1.5 (0.9)	138 ± 4.6 (0.9)	143 ± 4.0 (1.0)	151 ± 4.2 (1.0)	149 ± 7.0 (1.0)	247 ± 7.6 (1.0)	236 ± 10.3 (1.1)
0.75	42 ± 13.5 (1.4)	40 ± 4.5 (1.3)	132 ± 7.8 (0.9)	148 ± 3.6 (1.0)	164 ± 13.9 (1.1)	165 ± 13.6 (1.2)	245 ± 5.9 (1.0)	251 ± 5.1 (1.2)
1.50	39 ± 5.6 (1.3)	35 ± 3.6 (1.1)	144 ± 8.4 (1.0)	138 ± 7.6 (0.9)	183 ± 14.1 (1.2)	166 ± 3.1 (1.2)	250 ± 6.1 (1.0)	254 ± 5.6 (1.2)
2.50	40 ± 4.1 (1.4)	39 ± 6.0 (1.3)	138 ± 4.6 (0.9)	143 ± 6.1 (1.0)	173 ± 10.1 (1.2)	175 ± 7.6 (1.2)	251 ± 6.9 (1.0)	255 ± 7.2 (1.2)
Fraction B (flavonoids)								
0	29 ± 2,1	31 ± 2.7	141 ± 8.0	143 ± 4.0	148 ± 2.5	143 ± 5.0	239 ± 16.7	210 ± 11.1
0.12	32 ± 4.0 (1.1)	30 ± 5.9 (0.9)	142 ± 20.0 (1.0)	145 ± 7.9 (1.0)	144 ± 14.2 (0.9)	145 ± 4.2 (1.0)	223 ± 11.0 (0.9)	224 ± 6.0 (1.1)
0.38	40 ± 2.5* (1.4)	40 ± 8.7 (1.3)	132 ± 7.0 (0.9)	142 ± 3.1 (0.9)	140 ± 1.5 (0.9)	145 ± 6.5 (1.0)	198 ± 12.5 (0.8)	206 ± 11.4 (0.9)
0.75	42 ± 4.0** (1.4)	40 ± 7.5 (1.3)	138 ± 3.6 (0.9)	146 ± 5.1 (1.0)	145 ± 13.2 (0.9)	156 ± 12.4 (1.1)	213 ± 23.3 (0.9)	216 ± 17.1 (1.0)
1.50	50 ± 4.9** (1.7)	49 ± 6.4* (1.6)	156 ± 9.3 (1.1)	146 ± 5.9 (1.0)	155 ± 5.0 (1.0)	154 ± 6.5 (1.1)	246 ± 7.4 (1.0)	232 ± 13.6 (1.1)
2.50	57 ± 5.0**(1.9)	49 ± 4.6* (1.6)	158 ± 3.2 (1.1)	151 ± 7.8 (1.1)	136 ± 4.6 (0.9)	142 ± 7.9 (0.9)	250 ± 8.5 (1.0)	234 ± 11.0 (1.1)
Fraction C (alkaloids)								
0	43 ± 2.7	36 ± 2.0	131 ± 1.5	139 ± 2.5	163 ± 4.2	145 ± 9.5	242 ± 9.2	223 ± 11.9
0.12	37 ± 4.0 (0.9)	33 ± 5.0 (0.9)	133 ± 4.5 (1.0)	126 ± 6.6 (0.9)	165 ± 3.6 (1.0)	132 ± 3.1 (0.9)	241 ± 7.1 (0.9)	220 ± 4.5 (0.9)
0.38	43 ± 2.5 (1.0)	36 ± 6.0 (1.0)	146 ± 7.0 (1.1)	136 ± 9.1 (0.9)	178 ± 3.1 (1.1)	142 ± 2.0 (0.9)	256 ± 21.6 (1.1)	224 ± 9.9 (1.0)
0.75	44 ± 1.5 (1.0)	34 ± 11.1 (0.9)	136 ± 8.6 (1.0)	133 ± 4.5 (0.9)	183 ± 4.0 (1.1)	144 ± 4.6 (0.9)	294 ± 12.9 (1.2)	223 ± 14.1 (1.0)
1.50	72 ± 9.7* (1.7)	45 ± 3.1 (1.3)	152 ± 13.1 (1.2)	146 ± 4.0 (1.1)	189 ± 3.5 (1.1)	141 ± 10.0 (0.9)	310 ± 2.0 (1.3)	224 ± 11.2 (1.0)
Control +	608 ± 71.52	663 ± 35.4	711 ± 12.1	761 ± 28.0	914 ± 17.6	886 ± 49.3	1076 ± 57.1	1082 ± 72.9

MeOH: methanolic extract; 0 = negative control (DMSO—100 *μ*l/plate); Control +: Positive control.

^(a)^4-Nitro-*o*-phenylenediamine (10.0 *μ*g/plate); ^(b)^2-anthramine (1.25 *μ*g/plate); ^(c)^Sodium azide (1.25 *μ*g/plate); ^(d)^Mitomycin C (0.5 *μ*g/plate).

*P < .05, **P < .01 (ANOVA). The values in brackets are MI values.

**Table 2:** Mutagenic activity expressed as the mean and SD of the number of revertants/plate in bacterial strains TA98, TA100, TA97a and TA102 exposed to MeOH extract and fractions A, B and C of *I. suffruticosa*, at various doses, with (+S9) or without (−S9) metabolic activation.

Treatments mg/plate	Revertants/plate in *S. typhimurium* strains							
	TA98		TA97a		TA100		TA102	
	−S9^(a)^	+S9^(b)^	−S9^(a)^	+S9^(b)^	−S9^(c)^	+S9^(b)^	−S9^(d)^	+S9^(b)^
MeOH extract								
0	43 ± 2.7	36 ± 2.0	131 ± 1.5	139 ± 2.5	163 ± 4.2	145 ± 9.5	242 ± 9.2	223 ± 11.9
1.25	38 ± 4.6 (0.9)	41 ± 1.2 (1.1)	145 ± 3.1 (1.1)	138 ± 2.1 (0.9)	152 ± 4.0 (0.9)	141 ± 2.1 (0.9)	262 ± 2.5 (1.1)	219 ± 4.9 (0.9)
2.50	43 ± 4.5 (1.0)	40 ± 2.7 (1.1)	145 ± 2.1 (1.1)	142 ± 4.2 (1.0)	151 ± 2.1 (0.9)	147 ± 6.7 (1.0)	244 ± 7.6 (1.0)	225 ± 8.5 (1.0)
3.75	62 ± 2.5** (1.4)	44 ± 3.1 (1.2)	147 ± 4.0 (1.1)	145 ± 2.1 (1.0)	161 ± 2.7 (0.9)	146 ± 7.5 (1.0)	249 ± 6.7 (1.0)	242 ± 5.0 (1.1)
5.00	66 ± 1.5** (1.5)	44 ± 3.1 (1.2)	153 ± 5.0 (1.2)	142 ± 6.4 (1.0)	165 ± 3.1 (1.0)	152 ± 6.2 (1.0)	250 ± 6.1 (1.0)	234 ± 7.9 (1.0)
7.50	76 ± 5.9** (1.8)	45 ± 3.5 (1.2)	159 ± 7.8 (1.2)	151 ± 5.1 (1.0)	156 ± 1.5 (0.9)	149 ± 6.0 (1.0)	253 ± 3.5 (1.0)	237 ± 9.6 (1.1)
Fraction A (glycerolipids)								
0	43 ± 2.7	36 ± 2.0	131 ± 1.5	139 ± 2.5	148 ± 2.5	143 ± 5.0	239 ± 16.7	210 ± 11.1
0.12	46 ± 1.7 (1.1)	35 ± 2.0 (0.9)	131 ± 2.1 (1.0)	136 ± 3.5 (0.9)	159 ± 4.2 (1.1)	145 ± 3.0 (1.0)	251 ± 7.0 (1.1)	227 ± 7.6 (1.1)
0.38	47 ± 1.5 (1.1)	38 ± 2.5 (1.1)	134 ± 3.1 (1.0)	135 ± 3.1 (0.9)	160 ± 2.0 (1.1)	147 ± 3.1 (1.0)	260 ± 6.0 (1.1)	235 ± 5.0 (1.1)
0.75	50 ± 1.5 (1.2)	42 ± 1.5 (1.2)	139 ± 5.0 (1.1)	138 ± 1.2 (0.9)	163 ± 3.1 (1.1)	152 ± 2.1 (1.1)	264 ± 8.4 (1.1)	247 ± 5.0 (1.2)
1.50	55 ± 3.6 (1.1)	45 ± 1.6 (1.3)	143 ± 2.5 (1.1)	142 ± 2.1 (1.0)	170 ± 1.5 (1.1)	160 ± 2.0 (1.1)	265 ± 5.0 (1.1)	255 ± 5.0 (1.2)
Fraction B (flavonoids)								
0	43 ± 2.7	36 ± 2.0	131 ± 1.5	139 ± 2.5	148 ± 2.5	143 ± 5.0	239 ± 16.7	210 ± 11.1
0.12	45 ± 1.5 (1.0)	42 ± 2.0 (1.2)	142 ± 1.5 (1.0)	136 ± 2.0 (1.0)	149 ± 4.2 (1.0)	136 ± 3.1 (0.9)	244 ± 3.2 (1.0)	219 ± 8.1 (1.0)
0.38	52 ± 1.5* (1.2)	43 ± 4.5 (1.2)	142 ± 1.0 (1.0)	139 ± 1.5 (1.0)	152 ± 3.6 (1.0)	140 ± 2.5 (0.9)	249 ± 2.1 (1.0)	239 ± 6.1 (1.1)
0.75	63 ± 3.1** (1.5)	48 ± 1.2** (1.3)	144 ± 3.1 (1.1)	141 ± 2.1 (1.0)	153 ± 5.5 (1.0)	142 ± 2.1 (0.9)	248 ± 26.2 (1.0)	243 ± 5.0 (1.2)
1.50	81 ± 1.5** (1.9)	54 ± 2.5**(1.5)	149 ± 1.5 (1.1)	144 ± 3.2 (1.0)	158 ± 2.0 (1.1)	143 ± 2.0 (1.0)	272 ± 6.2 (1.1)	264 ± 4.7 (1.3)
Fraction C (alkaloids)								
0	43 ± 2.7	36 ± 2.0	131 ± 1.5	139 ± 2.5	148 ± 2.5	143 ± 5.0	239 ± 16.7	210 ± 11.1
0.12	46 ± 1.5 (1.1)	31 ± 1.7 (0.9)	142 ± 2.0 (1.0)	139 ± 1.2 (1.0)	151 ± 2.3 (1.0)	140 ± 2.7 (0.9)	255 ± 4.2 (1.1)	228 ± 5.7 (1.1)
0.38	50 ± 2.0 (1.2)	38 ± 1.5 (1.1)	146 ± 1.2 (1.1)	139 ± 3.1 (1.0)	153 ± 2.5 (1.0)	145 ± 3.0 (1.0)	269 ± 1.2 (1.1)	239 ± 4.2 (1.1)
0.75	69 ± 1.2* (1.6)	40 ± 1.5 (1.1)	152 ± 2.0 (1.0)	143 ± 3.1 (1.0)	165 ± 3.8 (1.1)	149 ± 2.1 (1.0)	278 ± 5.3 (1.2)	243 ± 5.9 (1.2)
1.50	85 ± 1.5**(1.9)	47 ± 1.2 (1.3)	152 ± 4.4 (1.2)	143 ± 1.5 (1.1)	180 ± 2.0 (1.2)	154 ± 2.5 (1.1)	306 ± 7.2 (1.3)	255 ± 4.7 (1.2)
Control +	466 ± 34.1	607 ± 30.3	703 ± 24.7	750 ± 55.7	955 ± 33.0	797 ± 32.1	1158 ± 65.8	1158 ± 49.4

MeOH: methanolic extract; 0 = negative control (DMSO—100 *μ*l/plate); Control +: positive control.

^(a)^4-Nitro-*o*-phenylenediamine (5.0 *μ*g/plate); ^(b)^2-anthramine (1.25 *μ*g/plate); ^(c)^Sodium azide (1.25 *μ*g/plate); ^(d)^Mitomycin C (0.5 *μ*g/plate).

*P < .05, **P < .01 (ANOVA). The values in brackets are MI values.

**Table 3:** Mutagenic activity expressed as the mean and SD of the number of revertants/plate in bacterial strain TA98 exposed to the compounds isolated from methanolic extract of *I. truxillensis* and *I. suffruticosa* at various doses, with (+S9) or without (−S9) metabolic activation.

Treatment (mg/plate)	TA98 (−S9)	TA98 (+S9)
Positive control	867 ± 1.4	991 ± 10.1
Negative control	30 ± 1.5	36 ± 4.0
Alkaloids		
*Indigo*		
0.125	38 ± 0.6 (1.3)	44 ± 2.7* (1.2)
0.25	42 ± 3.2 (1.4)	86 ± 5.3** (2.4)
0.50	45 ± 1.5 (1.5)	192 ± 2.5** (5.3)
0.75	50 ± 4.0* (1.7)	229 ± 3.6** (6.7)
1.00	57 ± 4.2** (1.9)	276 ± 5.3** (7.7)
*Indirubin*		
0.125	46 ± 2.7 (1.5)	36 ± 2.0 (1.0)
0.25	58 ± 3.8** (1.9)	36 ± 4.0 (1.0)
0.50	58 ± 3.0** (1.9)	38 ± 2.5 (1.1)
0.75	63 ± 1.7** (2.1)	43 ± 4.0 (1.2)
1.00	63 ± 3.6** (2.1)	46 ± 3.6 (1.3)

Negative control: DMSO (100 *μ*l/plate); Positive control: −S9: 4-nitro-*o*-phenylenediamine (10.0 *μ*g/plate); +S9: 2-anthramine (1.25 *μ*g/plate).

*P < .05, **P < .01 (ANOVA). The values in brackets are MI values.
